# Fe-MIL-101 exhibits selective cytotoxicity and inhibition of angiogenesis in ovarian cancer cells via downregulation of MMP

**DOI:** 10.1038/srep26126

**Published:** 2016-05-18

**Authors:** Jiaqiang Wang, Daomei Chen, Bin Li, Jiao He, Deliang Duan, Dandan Shao, Minfang Nie

**Affiliations:** 1 Yunnan Provincial Collaborative Innovation Center of Green Chemistry for Lignite Energy, Yunnan Province Engineering Research Center of Photocatalytic Treatment of Industrial Wastewater, The Universities’ Center for Photocatalytic Treatment of Pollutants in Yunnan Province, Key Laboratory of Medicinal Chemistry for Natural Resource, Ministry of Education, School of Energy, School of Chemical Sciences & Technology, Yunnan University, Kunming 650091, P.R. China

## Abstract

Though metal-organic frameworks (MOFs) have inspired potential applications in biomedicine, cytotoxicity studies of MOFs have been relatively rare. Here we demonstrate for the first time that an easily available MOF, Fe-MIL-101, possesses intrinsic activity against human SKOV3 ovarian cancer cells and suppress the proliferation of SKOV3 cells (IC_50_ = 23.6 μg mL^−1^) and normal mouse embryonic fibroblasts (BABL-3T3, IC_50_ = 78.3 μg mL^−1^) cells. It was more effective against SKOV3 cells than typical anticancer drugs such as artesunate (ART, IC_50_ = 96.9 μg mL^−1^) and oxaliplatin (OXA, IC_50_ = 64.4 μg mL^−1^), but had less effect on normal BABL-3T3 cells compared with ART (IC_50_ = 36.6 μg mL^−1^) and OXA (IC_50_ = 13.8 μg mL^−1^). Fe-MIL-101 induced apoptosis of human umbilical vein endothelial cells (HUVECs) via G0/G1 cell cycle arrest and decreased the mitochondrial membrane potential in HUVECs and induced apoptosis. Furthermore, Fe-MIL-101 exhibited stronger antiangiogenic effects in HUVEC cells than antiangiogenic inhibitor (SU5416) via downregulation the expression of MMP-2/9. Our results reveal a new role of Fe-MIL-101 as a novel, non-toxic anti-angiogenic agent that restricted ovarian tumour growth. These findings could open a new avenue of using MOFs as potential therapeutics in angiogenesis-dependent diseases, including ovarian cancer.

Ovarian cancer is the second most common gynaecological malignancy worldwide and the leading cause of death among gynaecologic neoplasms. The tumour microenvironment encompasses various stromal cells and extracellular matrix surrounding tumour cells and differs from the normal tissue environment^1^. Increasing evidence suggests that the tumour microenvironment also plays critical roles in tumour progression and metastasis, and new strategies for synergistic tumour therapeutics target both tumour microenvironment and tumour cells^1^. The tumour microenvironment comprises numerous signalling molecules and pathways that influence the angiogenic response^2^. Angiogenesis, the formation of new blood vessels from a pre-existing vascular network, is principally mediated by endothelial cell proliferation and migration. It provides oxygen and nutrients to actively proliferating tumour cells^3^, and is responsible for the development and metastasis of most types of tumours^4^. The pathogenesis of malignant tumours is mainly caused by the disruption of angiogenesis and the imbalanced endothelial remodelling and regression. Therefore, inhibition of angiogenesis is emerging as a promising new approach for cancer treatment^5^,^6^,^7^,^8^,^9^.

Matrix metalloproteinases (MMP), a family of zinc-binding proteins including the gelatinases MMP-2 and MMP-9, have been shown to play a central role in angiogenesis and tumour cell invasion and metastasis due to their ability to degrade the extracellular matrix^10^. Thus, downregulating MMP-2/9 expression or decreasing enzymatic activities in the tumour microenvironment is crucial in inhibiting angiogenesis, tumour invasion and metastasis^1^. As a result, MMP inhibitors have been recognized as drug candidates for anticancer therapeutics, and much effort has been made to design and develop molecules that inhibit MMP activity^5^,^10^.

In contrast to conventional approaches, nanotechnology presents novel opportunities to develop promising diagnostics and therapeutics tools for the treatment of cancer and many other diseases^11^,^12^. However, little evidence exists that nanomaterials themselves might possess intrinsic anticancer properties, and only a few nanoparticles’ intimate interactions with the tumour microenvironment were reported^13^,^14^,^15^,^16^,^17^. For example, Fe_3_O_4_ nanoparticles coated with piroctone olamine^18^, fullerene-based nanomaterial Gd@C_82_(OH)_22_^19^,^20^,^21^,^22^, hollow mesoporous carbon nanocapsules (HMCNs)^23^ and polysaccharide-based hydrogels^24^ have been found to exhibit inhibition of MMP activity. However, none have translated to clinical application due to the dose-limiting side effects following systemic administration of these pharmacological MMP inhibitors^24^.

Compared with nanostructures such as metallic-, dielectric-, magnetic-, liposomal-, and carbon-based structures with potential medical applications, metal-organic frameworks (MOFs) are currently eliciting much attention in materials science and biotechnology due to outstanding properties including crystalline open structures, extremely high surface areas, tunable pores, diverse structure and chemical functionality^25^,^26^. For instance, Fe-MIL-101_NH_2_, consisting of trimeric iron(III) octahedral clusters interconnected by 1,4-benzenedicarboxylate anions, has been reported as a nontoxic nanocarrier for the controlled delivery of several antitumoural and retroviral drugs^27^,^28^. Until now, the available toxicity information regarding MOFs have remained scarce and the contemporary use of MOFs for cancer treatment has been largely limited to serving as contrast agents for imaging techniques and carriers for drug delivery^28^,^29^,^30^,^31^,^32^. Few reports have directly used MOFs as anticancer agents.

Treating malignant tumours effectively with low toxicity remains a major challenge and is urgently required. Despite the intensive studies on the synthesis, characterization and possible applications, the biocompatibility and cytotoxicity of MOFs have rarely been investigated. Until now, the available toxicity information dealing with MOFs or coordination polymers has been limited^33^. For instance, the toxicological effects of three different iron carboxylate MOFs, MIL88A (fumarate), MIL88B (tetramethylterephthalate) and MIL100 (trimesate), was examined by Horcajada and colleagues both *in vitro* and *in vivo*^34^, and all three showed no observable toxicity. A recent study reported that these iron carboxylate MOFs were rapidly sequestered by the liver and spleen, biodegraded and directly eliminated through the urine or faeces without causing obvious toxicity^35^. The values of the half maximal inhibitory concentration (IC_50_) for the MIL-101_NH_2_BrBODIPY,1,3,5,7-tetramethyl-4,4-difluoro-8-bromomethyl-4-bora-3a,4a-diaza-*s*-indacene (BrBODIPY) coated MIL-101 on human HT-29 colon adenocarcinoma cells have also been reported^36^. Additionally, the *in vitro* toxicity of lanthanide-based MOFs was carried out in HT-29 and acute lymphoblastic leukaemia human cells, showing important cytotoxicity values^28^,^37^. Zinc MOFs exhibited a time and concentration dependent cytotoxicity in rat PC12 pheochromocytoma cells^38^. The CuBTC MOF associated drug 5-fluorouracil was extremely cytotoxic against human MCF-7 breast cancer adenocarcinoma cells and human HL60 acute promyelocytic leukaemia cells via apoptosis mechanisms^39^. The mechanisms of apoptosis induced by MOFs are largely unknown. Moreover, little is known about the interactions of MOFs with the tumour microenvironment, and the antiangiogenic properties and inhibition of MMPs of MOFs remain almost completely unknown. Therefore, more research is needed regarding the use of MOFs in cancer therapy applications.

Here we investigated the inhibitory effects of Fe-MIL-101 on SKOV3 human ovarian cancer, HeLa cervical cancer and A549 lung cancer cell line growth and the proliferation, migration and tube formation of human umbilical vein endothelial cells (HUVECs). We also studied the effect of Fe-MIL-101 on cell apoptosis and MMP-2 and MMP-9 expression in HUVECs and SKOV3 cells. This study evaluated the mechanism associated with the antitumour and antiangiogenic activity of Fe-MIL-101.

## Results and Discussion

### Characterization of Fe-MIL-101

Fe-MIL-101 was obtained from ferric hydroxide and terephthalic acid as previously described^25^. The textural properties of Fe-MIL-101 are summarized in the supplementary Table S1 and Fig. S1. The adsorption/desorption isotherm of Fe-MIL-101 is of type I indicating the presence of the microporous network, which possesses the Langmuir surface area (5400 m^2^g^**−**1^) and Brunauer–Emmer–Teller (BET) surface area (3710 m^2^g^**−**1^). The X-ray diffraction (XRD) pattern of Fe-MIL-101 is also shown in supplementary Fig. S2. The diffraction peaks all corresponded to the product synthesized by Skobelev. Together these results confirmed the proper synthesis of Fe-MIL-101.

### Effect of Fe-MIL-101 on cell growth

**Figure 1:**
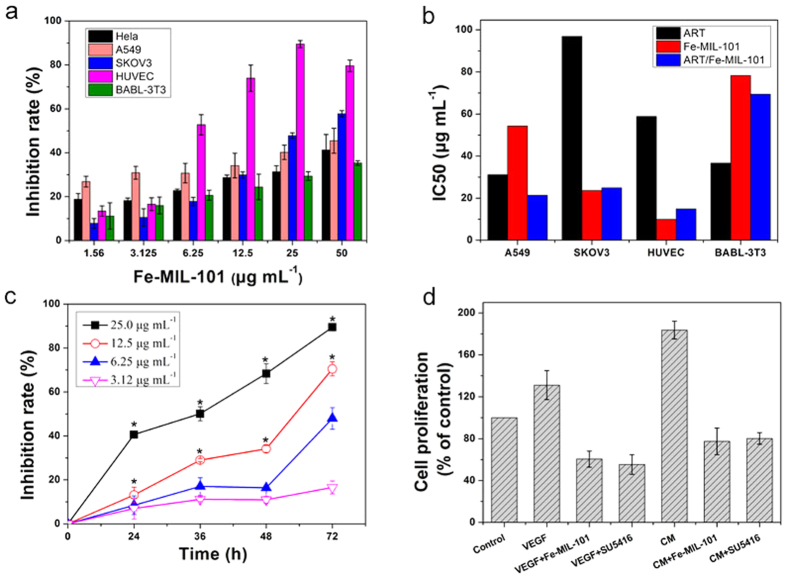
(**a**) Differential cytotoxicity of Fe-MIL-101 in cancer cells (HeLa, A549, SKOV3), human umbilical vein endothelial cells (HUVEC), and normal mouse embryonic fibroblasts cells (BABL-3T3) by MTT assay for 24–72 h. (**b**) The IC_50_ values of artesunate (ART), Fe-MIL-101 and ART/Fe-MIL-101 in cells for 72 h. (**c**) Inhibitory effect of Fe-MIL-101 on proliferation of HUVECs. (**d**) Proliferation of HUVECs treated with CM from SKOV3. *P < 0.05, vs. control.

The cytotoxicity of Fe-MIL-101 in four cell lines (HeLa, A549, SKOV3 and HUVEC cells) and control normal mouse embryonic fibroblast BABL-3T3 cells was evaluated by the MTT assay. Fe-MIL-101 exhibited concentration-dependent inhibition in HeLa, A549, SKOV3, HUVEC and BABL-3T3 cells with a half maximal inhibitory concentration (IC_50_) of 41.9, 74.6, 56.5, 33.5, and 91.2 μg mL^**−**1^ at 24 h, respectively (Table 1 and Fig. 1a). The IC_50_ for A549 cells was the highest among these cancer cells. These data show that Fe-MIL-101 is least active against A549 cells than other cells and demonstrate that Fe-MIL-101 has selective toxicity against different cancer cell lines.

Fe-MIL-101 also possessed cytotoxicity in a time-dependent manner in all cells except for HeLa and BABL-3T3 cells. The IC_50_ for A549, SKOV3 and HUVEC cells was reduced to 68.5, 37.3, and 24.5 μg mL^−1^, respectively, when treated with Fe-MIL-101 for 48 h. When treatment time was prolonged to 72 h, the IC_50_ value for A549, SKOV3 and HUVEC cells was 54.3, 23.6, and 9.9 μg mL^−1^, respectively, which was much lower than the values at 24 and 48 h.

Differential cytotoxicity is important because one of the greatest challenges facing chemotherapy is the inability of anticancer drugs to effectively distinguish between tumour cells and normal cells^40^. The IC_50_ of Fe-MIL-101 in BABL-3T3 cells was approximately 3.3- and 8-fold higher than that for SKOV3 cells and HUVEC cells, respectively. Fe-MIL-101 also has selective toxicity against HeLa, A549, and SKOV3 cancer cells and HUVEC cells and shows less toxicity against normal BABL-3T3 mouse embryonic fibroblasts cells (Fig. 1a). This suggests that Fe-MIL-101 may have a potential utility in the selective treatment of cancer cells.

Other Fe based MOFs, MIL-100 and MIL-88B, were also used to treat SKOV3 cells (Table 1). Fe-MIL-101 was more active against SKOV3 cells than Fe-MIL-100 and Fe-MIL-88B. Other Fe-based MOFs^34^ including MIL-100, MIL101_2CH_3_, MIL101_NH_2_, MIL88B and derivations (supplementary Table S2) exhibited low toxicity on HeLa cells with IC_50_ values ranging from 690 to 2500 μg mL^−1^. These values are much higher than that of Fe-MIL-101 (IC_50_ = 41.9 μg mL^−1^), indicating that the structure of iron terephthalic acid may play an important role. Therefore, the roles of Fe^3+^ and organic ligand (terephthalic acid, H_2_BDC) were also tested *in vitro*. The organic constitutive linker H_2_BDC was not toxic on HeLa cells with IC_50_ values 800 ± 20 μg mL^−1 ^34. In our study, the IC_50_ value of H_2_BDC for 24 h for SKOV3, BABL-3T3, and HeLa cells was 1353.9, 1111.6 and 1166.8 μg mL^−1^, respectively, suggesting that the organic linker H_2_BDC was not toxic. Iron is a component in haemoglobin and exists at approximately 22 μM in blood plasma, and shows little toxicity in the body^41^. Iron oxide nanoparticles are clinically approved as magnetic resonance imaging contrast agents, and *in vitro* assays have shown that these particles do not exhibit toxicity^34^. We also observed that the IC_50_ value of FeCl_3_•6H_2_O was above 1000 μg mL^−1^ for the above three mentioned cell lines. We also investigated the concentration of iron released from Fe-MIL-101 into medium by inductively coupled plasma-atomic emission spectrometry ICP-AES measurement. No detectable iron leaked into the medium at 37 °C after 72 h. These results suggest that Fe-MIL-101 instead of Fe^3+^ or organic ligand play key roles in cytotoxic activity and show that Fe-MIL-101 exhibited much higher cytotoxic activity against SKOV3 cells than other iron MOFs such as Fe-MIL-100 and Fe-MIL-88B (supplementary Table S2).

Other MOFs such as Tb-MOFs^35^ showed high cytotoxicity values against human HT-29 colon adenocarcinoma cells (IC_50_~10 μg mL^−1^) and Gd-MOFs^28^ also showed high cytotoxicity values against human acute lymphoblastic leukaemia cells (IC_50_~15 μg mL^−1^), which was due to the linkers’ antitumoural activity^34^. By contrast, the cytotoxicity of ZrMOFs UiO66^34^ (IC_50_ HeLa = 400 μg mL^−1^) is much lower than that of Fe-MIL-101.

Interestingly, Fe-MIL-101 exhibited higher cytotoxicity against SKOV3 cells than the anticancer drug carboplatin, for which IC_50_ is 54.6 μg mL^−1^ at 72 h^42^. Even compared with the positive controls artesunate (ART) and oxaliplatin (OXA), the inhibitory activity of Fe-MIL-101 was more effective than ART and OXA against SKOV3 and HUVEC cells, but only had a minor effect on HeLa, A549 and BABL-3T3 cells.

To achieve better anti-cancer effects, Fe-MIL-101 was used as carrier matrices for the ART delivery system. The amount of the drug laden in Fe-MIL-101 was measured by UV-vis spectroscopy and the ART contents were determined to be 0.70 g of ART per gram of the sample of Fe-MIL-101 (0.7 g/g material). Cytotoxic tests of ART/Fe-MIL-101 were also carried out against A549, SKOV3, HUVEC and BABL-3T3 cell lines (Table 1 and Fig. 1b). As shown in Fig. 1b, the IC_50_ values of ART were higher than that of ART/Fe-MIL-101 for A549, SKOV3 and HUVEC cells, suggesting that ART/Fe-MIL-101 exhibits better anti-proliferative effects than ART. However, except for A549 cells, the IC_50_ values of ART/Fe-MIL-101 on cells were similar to those of Fe-MIL-101 under the same condition. This implies that there is no significant difference between Fe-MIL-101 and ART/Fe-MIL-101. This could be attributed to the following reasons: the adsorption of ART led to a significant decrease in both surface areas, and pore volumes of Fe-MIL-101 indicated that ART was mainly adsorbed within the pores instead of on the external surface of Fe-MIL-101, whereas surface areas decrease from 3700 m^2^g^−1^ to 1760 m^2^g^−1^ and pore volumes decrease from 1.96 cm^3^g^−1^ to 0.93 cm^3^g^−1^ (Table S1, supplementary Fig. S3). Therefore, Fe-MIL-101 with very high cytotoxicity would contact SKOV3 cells first and cause cell death before the slow release of ART. The above results again confirm that Fe-MIL-101 is the major cause of cytotoxic activity in cancer and normal cells during this treatment time. More research is needed to obtain further insight into the reason why ART/Fe-MIL-101 did not enhance toxic effects compared with unloaded Fe-MIL-101.

To further evaluate the cytotoxic effect of the Doxorubicin (DOX) and DOX/Fe-MIL-101 in three cancer cell lines (HeLa, A549, and SKOV3 cells) and control normal mouse embryonic fibroblast BABL-3T3 cells by the MTT assay. As shown in supplementary Table S3, free DOX and DOX/Fe-MIL-101 also possessed cytotoxicity in a time-dependent manner in all cells. When treatment time was prolonged to 72 h, both free DOX and DOX/Fe-MIL-101 gave approximate IC50, indicating DOX/Fe-MIL-101 has similar cytotoxic effects against HeLa, A549, and SKOV3 cells than free DOX. However, the IC_50_ values of DOX/Fe-MIL-101 were higher than that of DOX for the normal cells, suggesting DOX/Fe-MIL-101 also shows less toxicity against normal BABL-3T3 mouse embryonic fibroblasts cells. This selective toxicity was consistent with Fe-MIL-101.

Endothelial cells are the main component of new capillary vessels, and endothelial cell proliferation plays a critical role in the formation of new capillaries^43^. As proliferation, migration and capillary tube formation of endothelial cells are critical steps in angiogenesis^44^, we investigated the effects of Fe-MIL-101 on the proliferation of HUVECs. As illustrated in Fig. 1c, treatment with Fe-MIL-101 (3.12–25 μg mL^−1^) for 24, 36, 48 and 72 h resulted in inhibition of cell proliferation in a concentration-dependent and time-dependent manner. The inhibitory rates of HUVEC proliferation at 24, 36, 48 h and 72 h were 40.6 ± 1.5%, 50.1 ± 3.2%, 68.4 ± 4.4% and 89.4 ± 1.6% for 25 μg mL^−1^ Fe-MIL-101, respectively. There were significant differences in concentrations higher than 6.25 μg mL^−1^ (*P < 0.01) (Fig. 1c). Previous studies demonstrated that ART exhibits anti-proliferation and anti-angiogenic activity as well as antitumour activity^45^. Interestingly, similar to the cytotoxicity study of Fe-MIL-101 and ART/Fe-MIL-101, Fe-MIL-101 was more effective than ART in inhibiting cell growth, and loading ART did not improve the cytotoxicity effect.

### The effect of Fe-MIL-101 on HUVEC proliferation in conditioned media (CM)

Vascular endothelial growth factor (VEGF) plays various functions in endothelial cells, including the induction of proliferation and differentiation^46^. VEGF increased the proliferation of HUVECs compared with controls (Fig. 1d). Moreover, CM from SKOV3 cells induced a significant increase in the proliferation of HUVECs to levels higher than treatment with VEGF. However, Fe-MIL-101 significantly inhibited cell proliferation under VEGF and CM treatment conditions. Furthermore, Fe-MIL-101 displayed a similar anti-proliferation effect compared with SU5416, a selective inhibitor of VEGF tyrosine kinase activity.

### Fe-MIL-101 induces apoptosis of HUVECs

**Figure 2:**
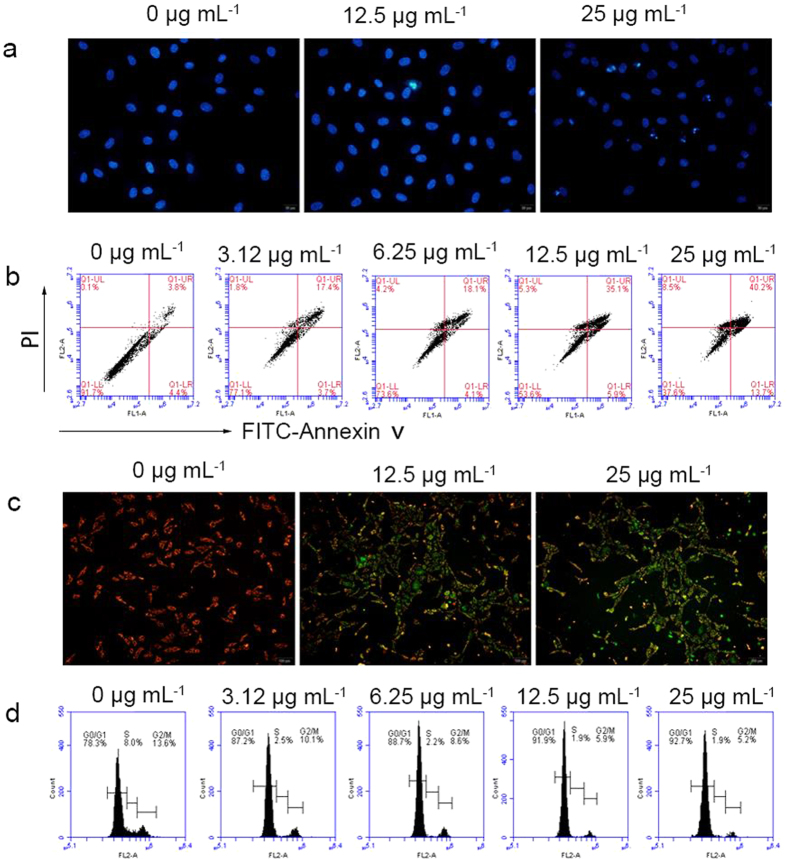
HUVEC apoptosis induced by Fe-MIL-101 for 48 h. (**a**) Hoechst 33342 staining of HUVEC cells. (**b**) After incubation with Fe-MIL-101 (0–25 μg mL^−1^) for 48 h, HUVECs were stained with FITC-Annexin-V and PI and analysed by flow cytometry. (**c**) Mitochondrial membrane potential was measured in HUVECs by JC1 and fluorescence probe staining. (**d**) HUVECs were treated with Fe-MIL-101 (0–25 μg mL^−1^) for 48 h and stained with propidium iodide to determine cellular DNA content by flow cytometry.

To determine the underlying mechanism of Fe-MIL-101 activity, the nuclear morphology of control and Fe-MIL-101 treated cells was observed by staining cell nuclei with Hoechst 33342 as previously described^47^. Apoptotic cells are generally characterized by condensation of chromatin and/or nuclear fragmentation. HUVEC cells were cultured and exposed to Fe-MIL-101 at concentrations of 12.5 and 25 μg mL^**−**1^ for 48 h. As shown in Fig. 2a, control cells, which were uniformly blue, were viable, whereas treated cells showed apoptosis, with nuclear shrinkage, chromatin condensation and cytoplasmic blebbing, and bright blue dots in the nuclei, representing nuclear fragmentation.

The FITC-Annexin-V and PI binding assay was used to further confirm Fe-MIL-101-induced apoptosis (Fig. 2b). In the dual parametric dot plots, the lower left quadrant represents the viable cell population (Annexin-V negative and PI negative), the upper right represents apoptotic cells undergoing secondary necrosis at the last stage or dead cells (Annexin-V and PI double positive), and the lower right represents the early stage apoptotic cell population (Annexin-V positive and PI negative). As the concentration of Fe-MIL-101 increased from 3.12–25 μg mL^−1^, the Annexin-V positive/PI negative cells increased from 3.7% to 13.7%, whereas the double positive cells increased from 17.4% to 40.2%. Increasing numbers of apoptotic cells progressed from the early stage to the late stage resulting in either death or secondary necrosis under Fe-MIL-101 at higher concentrations. This data confirms again that Fe-MIL-101 can effectively induce the apoptosis of HUVECs.

### Fe-MIL-101 decreases mitochondrial membrane potential of HUVECs

Mitochondria play a key role in the apoptotic pathway of cell death and changes in mitochondrial membrane permeability comprise the early events during apoptosis^48^. Mitochondrial membrane potential was measured using JC1 (5, 5′, 6, 6′-tetrachloro-1, 1′, 3, 3′-tetraethyl-imidacarbocyanine iodide) staining. Treatment of HUVEC cells with Fe-MIL-101 resulted in increased JC1 green fluorescence, indicating a decrease of mitochondrial membrane potential (Fig. 2c). These results indicate that Fe-MIL-101 induced apoptosis in HUVEC cells through the mitochondrial pathway. The adverse changes in mitochondrial function due to Fe-MIL-101, with possible association of intracellular reactive oxygen species (ROS) production, trigger the apoptosis process^49^. Fe^n+^ ions can generate ROS through the Fenton or Haber–Weiss reaction when in contact with cells^34^,^50^. It is noteworthy that redox active metals and ROS pathways alter the plasma membrane potential, provoking changes in cell cycle regulation^51^, or causing the membrane lipid peroxidation^52^, resulting in cellular death or apoptosis^34^.

### Fe-MIL-101 induces G0/G1 phase cell cycle arrest in HUVEC cells

The cell cycle is an important key step in angiogenesis^53^,^54^. We next performed flow cytometry to analyse the effects of Fe-MIL-101 on the cell cycle. Analysis of the cell cycle distribution of HUVEC cells indicated that Fe-MIL-101 treatment induced an increase in the percentage of G0/G1 phase cells and a reciprocal reduction in the percentage of S and G2/M phase cells (Table 2, Fig. 2d). Treatment with Fe-MIL-101 for 48 h led to a dose-dependent accumulation of cells in G0/G1 phase (64.5% in control cells versus 53.9% in cells treated with 25 μg mL^**−**1^ Fe-MIL-101; *P* < 0.05) and a decrease in the number of cells in S phase (*P* < 0.05) and G2/M phase (*P* < 0.05). We next evaluated apoptosis levels by flow cytometric Annexin V/PI assay. The results showed that 48 h incubation of cells with 3.12–25.0 μg mL^**−**1^ Fe-MIL-101 resulted in 21.1–53.9% apoptotic populations (Table 2), indicating that the apoptosis rate significantly increased with the increase of Fe-MIL-101 concentration (Fig. 2b). These data show that Fe-MIL-101 induced apoptosis on HUVECs via G0/G1 cell cycle arrest similar to nanoparticles such as [Gd@C_82_(OH)_22_]n^54^, gold nanoparticles (AuNP_40_)^55^, SDS-solubilized single-walled carbon nanotubes (SDS-SWCNTs)^56^ and silica nanoparticles^57^. For example, [Gd@C_82_(OH)_22_]n treatment of human MCF-7 breast cancer cells and human ECV304 umbilical vein endothelial cells induced G0/G1 phase arrest and induced cell apoptosis by downregulating CDK4, CDK6, cyclin E, cyclin D2, and Bcl-2 expressions and by upregulating p21 and Bax expressions^54^. AuNPs induced cell cycle arrest at the G0/G1 phase and induced apoptosis in A549 lung cancer cells^55^. SDS-SWCNTs caused cell cycle arrest at G0/G1 phase in normal rat NRK 52E kidney tubular cells^56^. Silica nanoparticles induced G1 phase arrest through upregulation of p53 and p21 in the rat embryonic ventricular myocardial cell line H9c2(2-1)^57^.

The effect of Fe-MIL-101on normal BABL-3T3 cells was also examined by flow cytometry for comparison. The results were shown in supplementary Fig. S4. The cell was mainly distributed G0/G1 phase (76%) in control cells by flow cytometry analysis. Treatment with different concentration of Fe-MIL-101 for 48 h not led to a change in G0/G1 phase. As well as Fe-MIL-101 has no effect on the distribution of cell S phase and G2/M phase, suggesting Fe-MIL-101 did not disrupt the cell cycle of normal cells. However, Fe-MIL-101 treatment induced an increase in the percentage of G0/G1 phase and a reciprocal reduction in the percentage of S and G2/M phase in human umbilical vein endothelial cells (HUVECs). Above results indicated that Fe-MIL-101 effectively induced HUVECs arrest at G0/G1 phase, but did not disrupt the cell cycle of normal cells, which maybe the cause of selectivity toxicity towards cancer cells but not the normal cells.

### Effects of Fe-MIL-101 on migration of HUVECs

**Figure 3:**
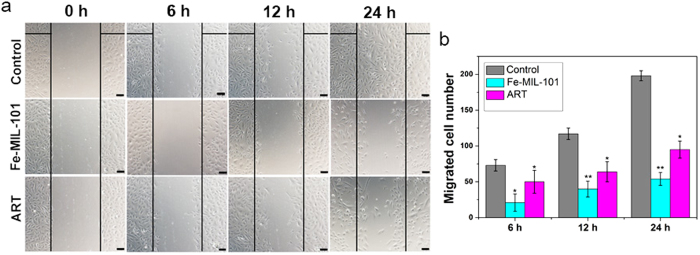
Fe-MIL-101 inhibits HUVEC cell migration. (**a**) Scratches were introduced by scraping the cell monolayer with a pipette tip. Scale bar: 100 μm. (**b**) Relative migration activity. Data represent the mean SD (n = 3). **P < 0.01, *P < 0.05 compared with control.

The migration of endothelial cells is an important step of angiogenesis. We performed wound-healing experiments and measured the amount of migrating cells to the wound area after HUVEC cells were treated with 12.5 μg mL^−1^ of Fe-MIL-101. ART, which inhibits migration of HUVECs^45^, served as a positive control (Fig. 3a). The migrating number of cells treated by Fe-MIL-101 was less than those treated with ART and the controls (Fig. 3b). In addition, Fe-MIL-101 inhibited HUVEC migration in a time-dependent manner. This effect was not due to cell death, because HUVECs did not show any morphological changes such as blebbing, cell shrinkage, nuclear fragmentation, chromatin condensation or chromosomal DNA fragmentation. HUVEC proliferation was also determined by MTT assay. Figure 1c shows that the inhibition rate was about 13.1% when HUVEC cells were treated with 12.5 μg mL^−1^ Fe-MIL-101 for 24 h, indicating that this concentration of Fe-MIL-101 was non-toxic over a short period and the inhibition of cell migration was not caused by the cytotoxicity of Fe-MIL-101. Together this indicates that Fe-MIL-101 possessed a stronger inhibitory effect on migration than control and ART groups, and this inhibitory effect was not due to the cytotoxicity of Fe-MIL-101. Fe-MIL-101 also inhibited SKOV3 cell migration in a time- and dose-dependent manner (supplementary Fig. S4). This suggests that Fe-MIL-101 has the potential to inhibit tumour and endothelial cell migration.

### Effects of Fe-MIL-101 on HUVEC tube formation

**Figure 4:**
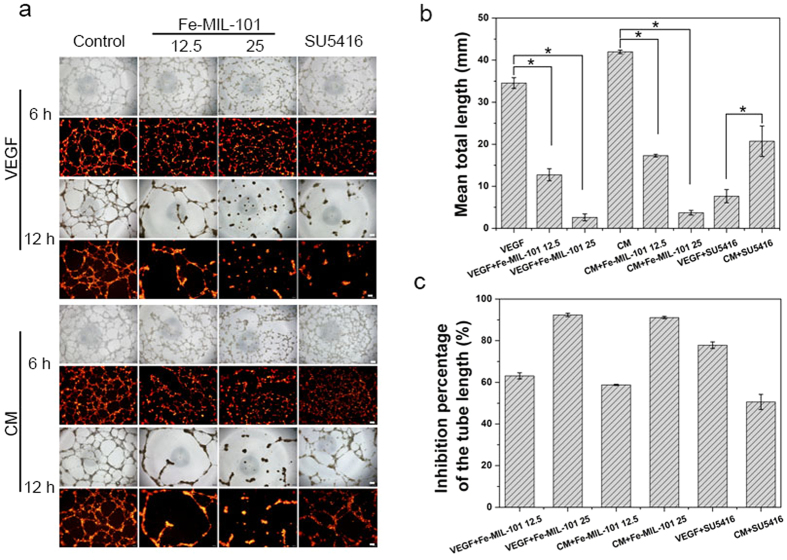
Fe-MIL-101 and SU5416 inhibit VEGF-induced or CM-induced tube formation of endothelial cells. (**a**) Dil-labeled HUVECs were inoculated on Matrigel-coated wells and treated with VEGF (10 ng mL^−1^) or CM prepared from SKOV3 cells in the presence or absence of either Fe-MIL-101 or 20 μM of SU5416. Scale bar: 200 μm. (**b**) Cumulative tube length in four fields/well (*P* < 0.05). (**c**) The inhibition percentage of the tube length in cells treated as indicated.

**Figure 5:**
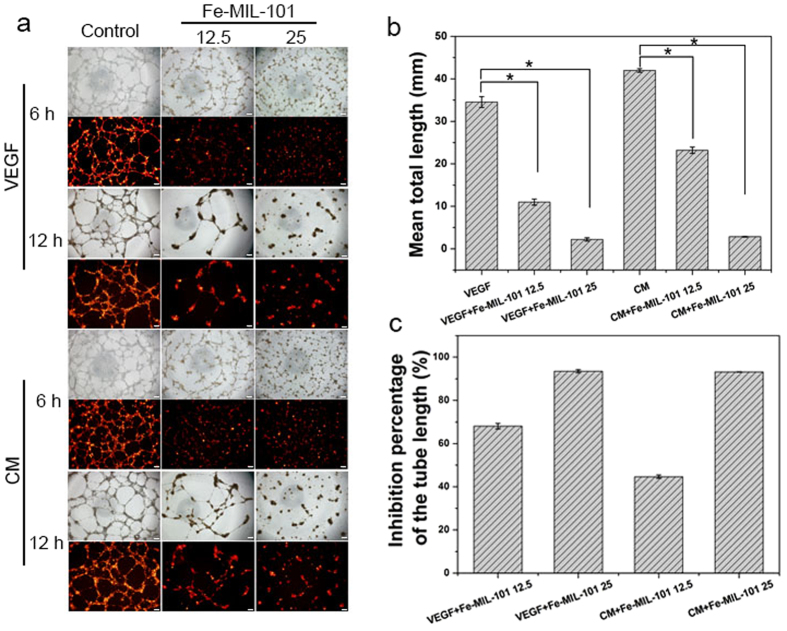
Tube formation is influenced by pre-treatment with Fe-MIL-101. (**a**) Capillary structure formation was observed in HUVECs incubated with Fe-MIL-101 (12.5, 25 μg mL^−1^) after 24 h. Scale bar: 200 μm. (**b**) Cumulative tube length in four fields/well (*P* < 0.05). (**c**) The inhibition percentage of the tube length in cells treated as indicated.

Tumour growth and proliferation depends on the formation of new blood vessels (vasculogenesis and angiogenesis) for the supply of oxygen and nutrients. VEGF induced endothelial cell morphogenic differentiation into capillary-like structures^58^ and CM collected from tumour cells increased endothelial cell proliferation, migration and tube-like structure formation *in vitro*^59^. Therefore, we assessed the effect of Fe-MIL-101 on new blood vessel formation *in vitro* of HUVECs treated with 10 ng mL^**−**1^ VEGF or CM collected from SKOV3 cells. As shown in Fig. 4a, HUVECs formed a robust and complete tube network within 12 h post-seeding. Tube formation of HUVEC was observed in all groups. The tubes of the CM treated group were longer than the VEGF treated group, which may be because CM contains some pro-angiogenic proteins, such as VEGFs, angiopoietin-2 and basic fibroblast growth factor (bFGF)^60^. Fe-MIL-101 (12.5 μg mL^**−**1^) partially abolished this process with an observed reduction of number and length of tube-like structures. Capillary tubes were completely disassembled in the presence of 25 μg mL^**−**1^ Fe-MIL-101. There was an approximately 60–90% reduction in the total tube length per field following 12.5 and 25 μg mL^**−**1^ Fe-MIL-101 treatment for 12 h (Fig. 4b,c), suggesting a decrease in a dose-dependent manner. Moreover, Fe-MIL-101 also had better antiangiogenic activity compared with the inhibitor SU5416 (20 μM). As shown in Fig. 4b, the length of tubes induced by 25 μg mL^**−**1^ Fe -MIL-101 was approximately three-fold shorter than that induced by SU5416 in the VEGF group and about 5.5-fold shorter than the length of tubes induced by SU5416 in the CM group. Similar results were observed in the group pre-treated with 25 μg mL^**−**1^ Fe-MIL-101 (Fig. 5a–c). In the presence of VEGF, tube formation was inhibited by 93%, which showed a comparable effect in the presence of CM. AgNPs^9^ (80 μg mL^**−**1^) inhibited about 80% tube formation of bovine retinal endothelial cells on Matrigel. HMCNs^23^ effectively inhibited the formation of tubes induced by VEGF. Based on the inhibition percentage equation, Fe-MIL-101 inhibited more than 90% tube formation of HUVECs (Figs 4c and 5c), suggesting that Fe-MIL-101 exhibited better antiangiogenic activity compared with AgNPs and HMCNs. These data show that Fe-MIL-101 could serve as a potential candidate for anti-angiogenic therapy.

### Expression of MMP-2 and MMP-9 in SKOV3 and HUVEC cells

**Figure 6:**
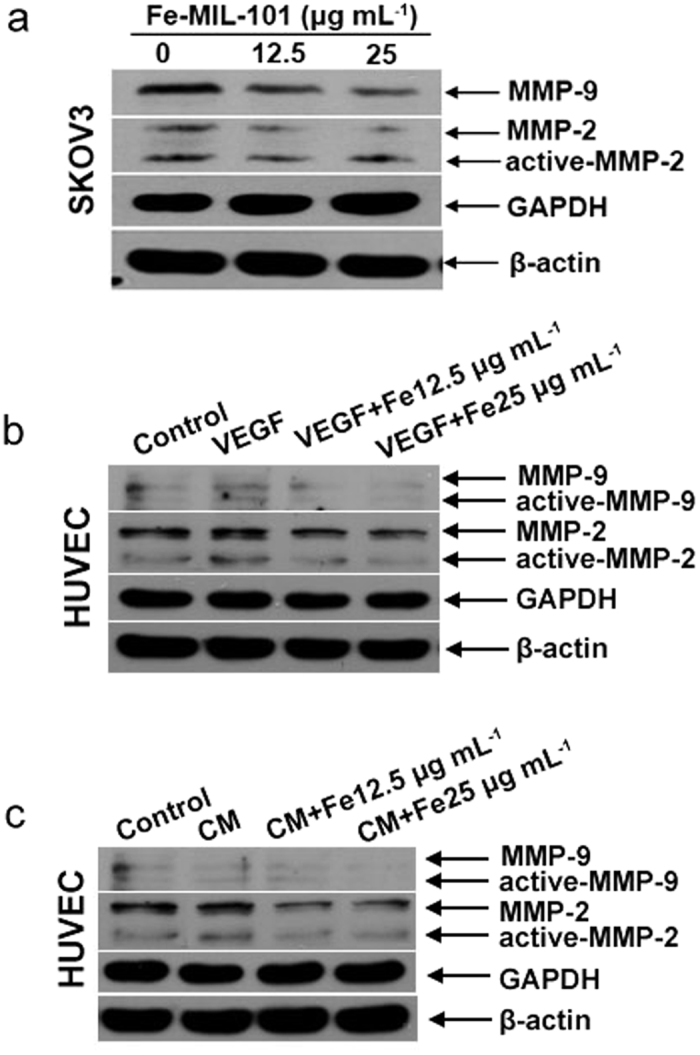
Inhibitory effects of Fe-MIL-101 on the expressions of MMP-2/9. Inhibitory effects of Fe-MIL-101 on the expressions of MMP-2/9 in SKOV3 cells (**a**), in HUVEC cells line induced by VEGF (**b**), and under induction of CM (**c**).

MMPs appear to play an important role in the process of tumour progression and angiogenesis of endothelial cells. Thus, we investigated MMP-2/9 protein expression in SKOV3 and HUVEC cells. MMPs are secreted as pro-enzymes that become active when cleaved. Western blot analysis showed that the total protein expression of MMP-2 (MMP-2 and active-MMP-2) and MMP-9 markedly decreased in SKOV3 cells upon Fe-MIL-101 treatment in a dose-dependent manner (Fig. 6a). Analyses of MMPs in HUVECs showed that VEGF and CM can upregulate the expression of MMP-2, MMP-9 and their active forms (Fig. 6b,c). These results are consistent with previous studies^61^,^62^. It is also interesting to note that treatment with Fe-MIL-101 significantly inhibited VEGF or CM-induced upregulation of MMP expression and activities. Moreover, MMP-2/9 levels in SKOV3 cells were significantly diminished in a dose-dependent manner upon treatment with Fe-MIL-101 for 24 h. Recent studies have shown that MMP-2 and MMP-9 are critical in the progression of angiogenesis. For example, the fullerene-based nanoparticle Gd@C_82_(OH)_22_ and hollow mesoporous carbon nanocapsules (HMCNs) serve as potent antiangiogenesis inhibitors through downregulating multiple angiogenic factors including MMP-2/9^21^,^23^. Compared with Gd@C_82_(OH)_22_, HMCN nanoparticles, the synthesis of Fe-MIL-101 is more simple. Moreover, Fe-MIL-101 possesses better activity of inhibition of MMP-2/9 than HMCN nanoparticles. Together the above results reveal that proliferation and migration of tumour cells and HUVECs were inhibited in the presence of Fe-MIL-101. Numerous gene products including urinary plasminogen activator, matrix metalloproteinases (MMP-2, MMP-9) and cyclooxygenase 2 and other chemokines regulate cell migration, invasion and tube formation processes^63^. Our results show that the expressions of MMP-2 and MMP-9 proteins in SKOV3 and HUVEC cells incubated with Fe-MIL-101 are significantly downregulated in a concentration-dependent manner, suggesting that Fe-MIL-101 exhibits significant inhibitory effect on the metastasis of metastatic cancer cells by downregulating the expressions of metastasis-related proteins.

## Conclusions

Fe-MIL-101 was found to elicit anti-proliferative effect on HeLa, A549, SKOV3 cancer cells and HUVECs and showed less toxicity against BABL-3T3 normal mouse embryonic fibroblast cells. Fe-MIL-101 may have a potential utility to distinguish effectively between HeLa, A549, SKOV3 tumour cells and normal cells. Moreover, Fe-MIL-101 effectively induced apoptosis of HUVECs through the mitochondrial pathway and caused G0/G1 cell cycle arrest, but has no effect on cell cycle of normal BABL-3T3 cells. In addition, Fe-MIL-101 suppressed angiogenesis processes *in vitro*. Fe-MIL-101 has the potential to inhibit tumour and endothelial cell growth and migration mainly through downregulation of MMP**-**2/9 protein expression. Therefore, the antitumour and anti-angiogenic efficacy of Fe-MIL-101 suggest that Fe-MIL-101 may be a promising novel candidate for cancer chemotherapy and our results help provide new insights into the application of MOFs.

## Methods

### Chemicals and instrumentation

Terephthalic acid (H_2_BDC, 99%), ferric chloride hexahydrate (FeCl_3_•6H_2_O, 99%), ethanol (99.5%), and *N, N* dimethylformamide (DMF, 99.9%) were purchased from Alfa Aesar (Ward Hill, MA) and used for synthesis. All organic solvents were of analytical grade. Artesunate (C_19_H_28_O_8_, 98%, MW = 384.4) was purchased from Guiling Pharmaceutical Co. Ltd., (Guangxi, China). Oxaliplatin was purchased from Jiangsu Aosaikang Pharmaceutical Co., Ltd., (Nanjing, China). Doxorubicin hydrochloride (8S,10S) -10-(4-amino-5-hydroxy-6-methyl-tetrahydro-2H-pyran-2-yloxy) -,8,11 – trihydroxy -8- (2-hydroxyacetyl)-1-methoxy -7,8,9,10 -tetrahydrotetracene -5, 12-dione were purchased from Alfa Aesar (Ward Hill, MA). The Annexin-V/PI detection apoptotic kit, JC1 lipophilic cation (5, 5′, 6, 6′ tetrachloro 1, 1′, 3, 3′ tetraethyl benzimidazol carbocyanine iodide), and mitochondrial membrane potential detection kit were from Beyotime Institute of Biotechnology (Jiangsu, China).

Fourier transform infrared measurements were performed on a Nicolet 8700 instrument. X-ray powder diffraction (XRD) experiments were conducted on a D/max-3B spectrometer with Cu Kα radiation. Pore size distributions, BET surface areas and pore volumes were measured by nitrogen adsorption/desorption measurements using a Micromeritics Tristar II Surface area and porosity analyser. Prior to the analysis, the samples were degassed at 90 °C for 1 h. Inductively coupled plasma-atomic emission spectrometry (ICP-AES) analysis was used to determine the contents of Fe^3+^ released from Fe-MIL-101. ICP-AES measurement was carried out with a Shimadzu ICPS-1000IV model. Fluorescence spectroscopy measurements were carried out using a F-7000 FL Spectrophotometer. Cells were analysed using a FACSCalibur flow cytometer (Becton Dickinson & Co., Franklin Lakes, NJ) and an Olympus IX73 microscope.

### Synthesis of Fe-MIL-101

MOF Fe-MIL-101 was synthesized with ferric hydroxide and terephthalic acid according to the literature^1^. Briefly, FeCl_3_•6H_2_O (0.675 g, 2.45 mmol) and H_2_BDC (0.206 g, 1.24 mmol) were added slowly into DMF (15 mL) solution. The mixture was stirred for 10 min at room temperature, and then transferred into a Teflon-lined stainless steel autoclave and heated at 110 °C for 20 h. The resulting brown solid was filtered off, and the raw product was purified by washing in hot ethanol (70 °C, 3 h), filtered off, and dried in an oven (70 °C, 30 min). The particles were isolated by centrifuging and washed with DMF and ethanol to remove any unreacted starting materials.

### Incorporation of artesunate and doxorubicin

Artesunate (ART) and Doxorubicin (DOX) were loaded in Fe-MIL-101 according to the literature^27^. Briefly, 20 mg Fe-MIL-101 and 40 mg ART were added in 10 mL acetone solution and stirred for 72 hours. The precipitate was separated by centrifuging at 13 000 rpm and washed several times with acetone. Then, the ART/Fe-MIL-101 was prepared and dried under vacuum during 3 days. The ART was quantified by was estimated by UV-Vis spectroscopy at 238 nm. The ART contents were determined to be 0.70 g of ART per gram of the sample of Fe-MIL-101 (0.70 g/g material).

12.5 mg Fe-MIL-101 was added in 5 mL of a solution of DOX in ethanol (5 mg mL^−1^) under stirring at room temperature for 72 hours. The resulting nanoparticles were then purified by centrifugation and washed several times with ethanol. This process was repeated until no fluorescence signal from free, non-encapsulated drug was detected in the supernatant solution. Then, the DOX/Fe-MIL-101 was prepared and dried under vacuum during 3 days. DOX was quantified by fluorescence spectroscopy (emission maximum at 551 nm when excited at 472 nm). The DOX contents were determined to be 0.43 g of DOX per gram of the sample of Fe-MIL-101 (0.43 g/g material).

### Cell culture

BABL-3T3 mouse embryonic fibroblasts cells, A549 human lung adenocarcinoma cells, HeLa human cervical cancer cells, and SKOV3 human ovarian cancer cells were purchased from the American Type Culture Collection (ATCC, Manassas, VA, USA). The BABL-3T3 cell line was cultured in DMEM (high glucose) and other cells were grown in DMEM (low glucose) containing 10% fetal bovine serum. HUVECs were isolated from term umbilical cord veins using collagenase and cultured in DMEM supplemented with 20% fetal bovine serum. All cell lines were grown at 37 °C in a humidified 5% CO_2_ atmosphere. HUVEC cells were used within 6 passages.

### MTT assays

SKOV3, A549, HeLa, BABL-3T3 and HUVEC cells were plated in 96-well plates at a density of 1 × 10^4^ cells per well. After 24 h of culture in the normal growth medium, cells were exposed to various concentrations (1.56–25 μg mL^**−**1^) of Fe-MIL-101 for 24, 48 and 72 h. Cells were incubated with 5 μg mL^**−**1^ MTT solution for 4 h, and 150 μL DMSO was added for dissolving crystals. Absorbance at 490 nm was determined using a Spectra Max 190 microplate spectrophotometer (Molecular Devices Corporation, USA). Cell numbers were obtained as absorbance values. The results were expressed as IC_50_ values. The experiment was repeated at least three times.

### Conditioned media sample preparation and the effect of Fe-MIL-101 on HUVEC proliferation in conditioned media

Conditioned media (CM) was collected from P6 SKOV3 cells. Cells at 80–90% confluence were washed with PBS three times and incubated with fresh DMEM without FBS medium (1 ml of medium per 9 cm^2^ of growth area) for 24 h at 37 °C. Media were then centrifuged (600 g, 10 min, 4 °C) and stored at **−**80 °C for further experiments.

Proliferation was assessed using the MTT assay in 96-well plates. HUVECs were incubated for 24 h in 10% FBS medium, and then replaced with 2% FBS media and incubated overnight before treatment. VEGF (10 ng mL^−1^) and SU5416 (20 μM) were used as positive and negative control groups, respectively. Cells were exposed to of Fe-MIL-101 (25 μg mL^−1^). After 24 h, 5 μg mL^−1^ MTT reagent was added in solution for 4 h, and 150 μL DMSO was added into plates for dissolving crystals. Absorbance at 490 nm was determined as described above.

### Apoptosis assay by Hoechst 33342 staining

HUVEC cells were seeded at a density of 2 × 10^5^ cells/well on the surface of a cover slip in a 6-well plate in 2 mL medium containing 10% FBS. After 24 h, cells were treated with Fe-MIL-101 (12.5 or 25 μg mL^**−**1^) and incubated for 48 h. Cells were washed with ice-cold PBS, and fixed with 4% paraformaldehyde for 10 min. The cells were incubated with Hoechst 33342 for 15 min at room temperature after washing three times with 2 mL of PBS. The cover slips were mounted on glass slides and the cells were analysed using a confocal fluorescence microscopic system (Olympus IX73, Japan).

### Flow cytometry analysis of apoptotic and necrotic cells

After chemical treatment, cells (1 × 10^6^) were harvested, washed with PBS, fixed with 70% ethanol, and maintained at 4 °C for at least 12 h. Pellets were stained with the fluorescent probe solution containing 5 μg mL^**−**1^ PI and 1 μg mL^**−**1^ FITC-Annexin-V in PBS on ice in dark for 15 min. The fluorescence emission was measured at 490 nm using 488-nm excitation by a FACSCalibur flow cytometer (Beckman Dickinson & Co., Franklin Lakes, NJ). A minimum of 1 × 10^4^ cells was analysed.

### Mitochondrial membrane potential assay

HUVECs were treated with Fe-MIL-101 for 48 h in 24-well plates and washed three times with cold PBS. Cells were incubated with 5 μg mL^**−**1^ of JC1 for 20 min in culture medium at 37 °C in the dark. Cells were imaged under a fluorescence microscope (Olympus IX73, Japan).

### Flow cytometry analysis of cell cycle

HUVECs were seeded into 6-well plates at a density of 1 × 10^6^ cells per well and incubated for 24 h in DMEM supplemented with 10% FBS at 37 °C and 5% CO_2_. The medium was removed and replaced with medium (final DMSO concentration, 0.05% v/v) containing Fe-MIL-101 (3.12, 6.25, 12.5, or 25 μg mL^**−**1^). After incubation for 48 h, the cell layer was trypsinized, washed with PBS and fixed with 70% ethanol. Next, 20 μL of RNAse (0.2 μg mL^**−**1^) and 20 μL of propidium iodide (0.02 μg mL^**−**1^) were added to the cell suspensions and they were incubated at 37 °C for 30 min. Samples were analysed with a FACSCalibur flow cytometer (Becton Dickinson & Co., Franklin Lakes, NJ). A minimum of 1 × 10^4^ cells was analysed.

### HUVEC migration assay

After cells were grown to confluence in a 6-well plate, cell monolayers were scratched with a 200-μL pipette tip. Cells were washed twice and the medium was replaced by fresh CM in DMEM. After 24 h under various conditions, the number of migrated cells into the scratch area was quantified using an inverted microscope fitted with a digital camera (Olympus IX 73, Japan).

### Tube formation assay

Tubular network formation assay was performed as described by Mirshahi^64^. Briefly, 50 μL Matrigel was added to each well of 96-well plates and the plates were incubated at 37 °C for 30 min for hardening. Then, 100 μL HUVEC suspensions (2 × 10^5^ cells per mL), different doses of Fe-MIL-101, VEGF (10 ng mL^**−**1^) and CM were seeded on the Matrigel-coated plates for 12 h. In the other group, HUVECs pre-treated with Fe-MIL-101 (12.5, 25 μg mL^**−**1^), VEGF (10 ng mL^**−**1^) and CM for 12 h were prepared by trypsinization, washed once with growth medium, and re-suspended at 2 × 10^5^ cells per mL in growth medium. Cells (100 μL) were gently added to the Matrigel-coated plates and incubated at 37 °C for 12 h. Tubular network formation was quantified by measuring the length of tubules in randomly chosen five fields under a fluorescence microscope (Olympus IX73, Japan). The total length of a tube-like network in each photograph was measured using Adobe Photoshop TM software. The inhibition percentage (%) of a sample was calculated by the following equation: inhibition percentage = (LC**−**LS) /LC × 100%, where LC is the average total capillary length of three control wells and LS is the average total capillary length of the three sample-treated wells.

### Western blot

Cells were washed with cold PBS and resuspended in lysis buffer (20 mM Tris-HCl, 150 mM NaCl, 1% Triton X-100, SDS, 10% β-mercaptoethanol, 1 mM PMSF, EDTA and leupeptin) for 1 h on ice. The lysates were centrifuged at 4 °C for 15 min (14,000 g). Proteins were separated by 10% SDS-PAGE and transferred onto PVDF membranes by standard procedures. Membranes were incubated sequentially with blocking agents (5% non-fat milk in PBS-Tween), primary antibodies (MMP-2/9 1:200, β-actin 1:1000, GAPDH 1:3000) and HRP-conjugated secondary antibodies. The signal was detected by chemiluminescence agents.

### Statistical analysis

All values are expressed as the mean ± S.D. and the significant levels between two groups were assessed by Student’s *t*-test. Results were considered statistically significant at 95% confidence interval (i.e., *P* < 0.05). All figures and graphical readings shown were obtained from at least three independent experiments.

## Additional Information

**How to cite this article**: Wang, J. *et al*. Fe-MIL-101 exhibits selective cytotoxicity and inhibition of angiogenesis in ovarian cancer cells via downregulation of MMP. *Sci. Rep*. **6**, 26126; doi: 10.1038/srep26126 (2016).

## Supplementary Material

Supplementary Information

## Figures and Tables

**Table 1:** Comparison of IC_50_ (μg mL^−1^) of five cell lines by anticancer cell drugs and Fe-MIL-101.

Cell line	Time (h)	Fe-MIL-101	OXA	ART	ART/Fe-MIL-101
HeLa	24	41.9	84.8	126.5	–
	48	50.1	28.5	62.7	–
	72	67.8	20.0	43.6	–
A549	24	74.6	219.8	160.7	128.3
	48	68.5	128.3	66.0	55.8
	72	54.3	27.0	31.1	21.4
SKOV3	24	56.5	241.5	280.8	42.9
	48	37.3	120.8	121.1	25.1
	72	23.6*	64.6	96.9	24.9
HUVEC	24	33.5	98.0	145.7	34.0
	48	24.5	31.3	87.9	22.8
	72	9.9*	20.9	58.8	14.9
BABL-3T3	24	91.2	77.8	118.2	91.6
	48	78.8	34.2	52.4	74.0
	72	78.3	13.8	36.6	69.4

**Table 2:** Apoptosis of HUVEC cells when treated with Fe-MIL-101 for 48 h detected by flow cytometry.

Conc. μg mL^−1^	Distribution of cell cycle (%)			Apoptosis rate (%)
	G0/G1	S	G2/M	
0	78.5 ± 1.7	18.1 ± 1.1	13.3 ± 1.1	7.4
3.12	87.4 ± 2.3	5.5 ± 2.3	9.9 ± 2.1	21.1
6.25	88.8 ± 2.2	2.3 ± 1.6	8.4 ± 1.4	22.2
12.5	91.9 ± 2.7*	1.9 ± 1.6*	5.9 ± 1.6	40.0*
25	92.8 ± 2.5*	1.9 ± 1.0*	5.1 ± 2.1	53.9*
