# Supplementary material for: Fe-MIL-101 exhibits selective cytotoxicity and inhibition of angiogenesis in ovarian cancer cells via downregulation of MMP
Source: Sci Rep. 2016 May 18;6:26126. doi: 10.1038/srep26126 (PMC4870622; doi:10.1038/srep26126)
Supplement: Supplementary Information [file srep26126-s1.doc]

Supplementary information

**Fe-MIL-101 exhibits selective cytotoxicity and inhibition of angiogenesis in ovarian cancer cells via downregulation of MMP**

Jiaqiang Wang,* Daomei Chen, Bin Li,* Jiao He, Deliang Duan, Dandan Shao and Minfang Nie

Yunnan Provincial Collaborative Innovation Center of Green Chemistry for Lignite Energy, Yunnan Province Engineering Research Center of Photocatalytic Treatment of Industrial Wastewater, The Universities’ Center for Photocatalytic Treatment of Pollutants in Yunnan Province, Key Laboratory of Medicinal Chemistry for Natural Resource, Ministry of Education, School of Energy, School of Chemical Sciences & Technology, Yunnan University, Kunming 650091, P.R. China.

Fax: (+) 86 871 65031567
E-mail: [**jqwang@ynu.edu.cn**](mailto:jqwang@ynu.edu.cn)**;** [**libin36@ynu.edu.cn**](mailto:libin36@ynu.edu.cn)

**Table S1.** Textural properties of Fe-MIL-101.

|  | S_Langmuir_ (m^2^ g^−1^) | S_BET_ (m^2^ g^−1^) | V_P_ (cm^3^ g^−1^) |
| --- | --- | --- | --- |
| Fe-MIL-101 | 5400 | 3710 | 1.96 |
| ART/Fe-MIL-101 | 2553 | 1766 | 0.93 |

Note: the samples were degassed at 90°C for 1h.


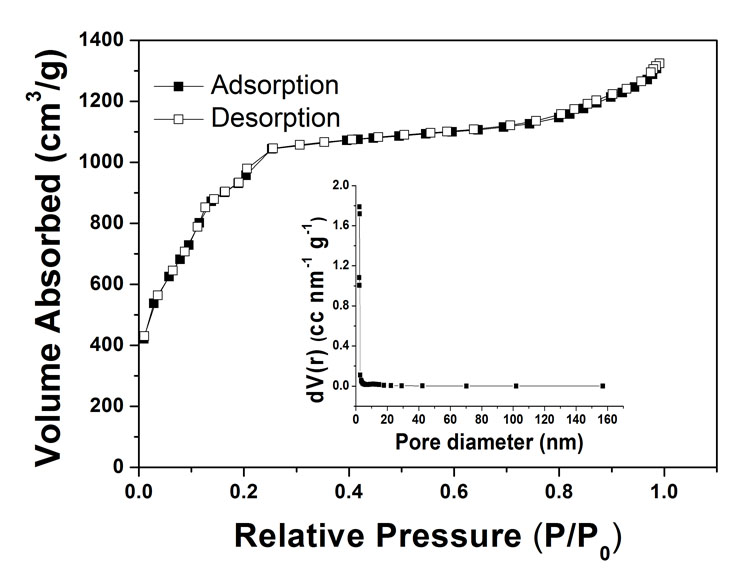


Fig.S1 N_2_ adsorption/desorption isotherm and the BJH pore-size distribution (inset) of Fe-MIL-101.


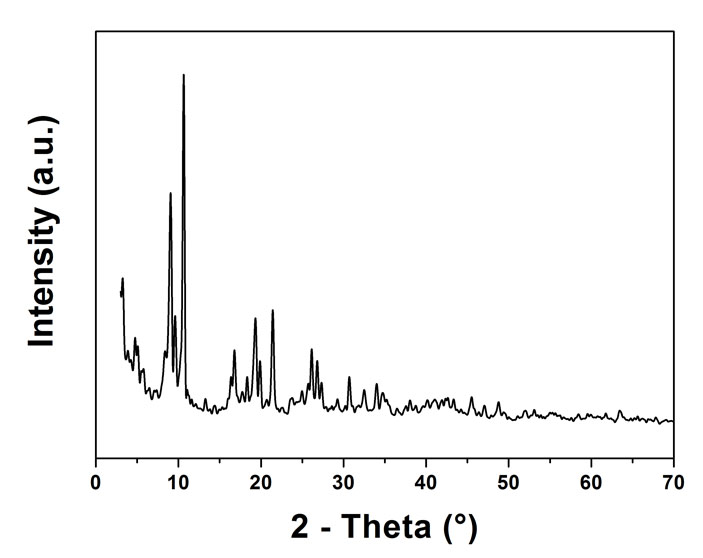


Fig.S2 Powder XRD patterns of Fe-MIL-101.


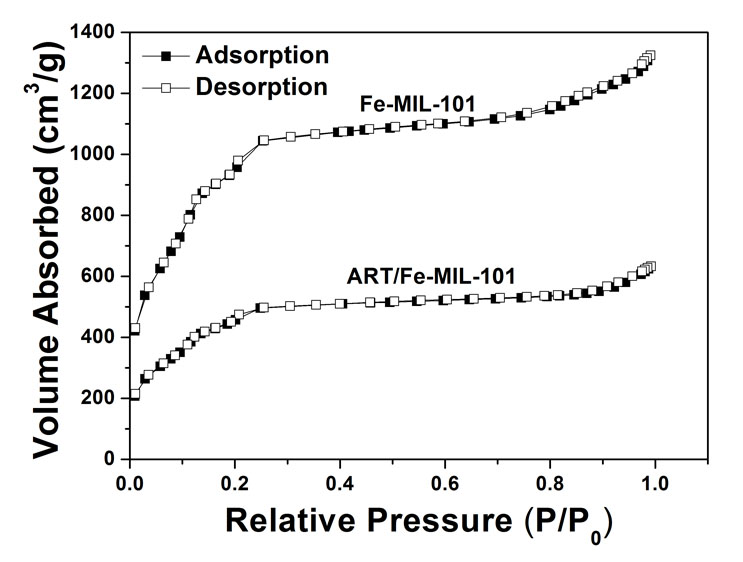


Fig.S3 N_2_ adsorption/desorption isotherm of before and after absorption of artesunate (ART).

**Table S2.** Inhibition (IC_50_) of cell lines by different MOFs materials.

| MOF NPs | Cell line | Time (h) | IC_50_ (μg mL^-1^) | Refs |
| --- | --- | --- | --- | --- |
| Fe-MIL- 100 | Hela | 24 | 1100±150 | 1 |
| Fe-MIL-88B | Hela | 24 | 1260±80 | 1 |
| Fe-MIL-88A | Hela | 24 | 15±5 | 1 |
| Fe-MIL-101_2CH3 | Hela | 24 | >2500 | 1 |
| Fe-MIL-101_NH_2_ | Hela | 24 | >1000 | 1 |
| UiO-66 | Hela | 24 | 400.0 | 1 |
| Fe-MIL-101_NH_2_BrBODIPY | HT-29 | 72 | 46.0 | 1, 2 |
| Tb-MOF | HT-29 | 72 | 10.0 | 1, 3 |
| Gd-MOF | ALL | 72 | 15.0 | 1, 4 |
| Fe-MIL-100 | SKOV3 | 24 | 80.8 | This work |
| Fe-MIL-100 | SKOV3 | 48 | 88.6 | This work |
| Fe-MIL-88B | SKOV3 | 24 | 252.0 | This work |
| Fe-MIL-88B | SKOV3 | 48 | 69.1 | This work |

**Table S3.** Comparison of IC_50_ (μg mL^−1^) of four cell lines by doxorubicin (DOX), DOX/Fe-MIL-101 and Fe-MIL-101.

| Cell line | Time (h) | Fe-MIL-101 | DOX | DOX/Fe-MIL-101 |
| --- | --- | --- | --- | --- |
| HeLa | 24 | 41.9 | 4.9 | 6.4 |
|  | 48 | 50.1 | 4.3 | 5.6 |
|  | 72 | 67.8 | 1.7 | 2.3 |
| A549 | 24 | 74.6 | 6.4 | 7.3 |
|  | 48 | 68.5 | 4.0 | 4.1 |
|  | 72 | 54.3 | 1.5 | 2.8 |
| SKOV3 | 24 | 56.5 | 4.7 | 9.3 |
|  | 48 | 37.3 | 3.1 | 7.9 |
|  | 72 | 23.6 | 1.5 | 2.6 |
| BABL-3T3 | 24 | 91.2 | 3.8 | 14.2 |
|  | 48 | 78.8 | 2.0 | 10.4 |
|  | 72 | 78.3 | 1.4 | 8.2 |


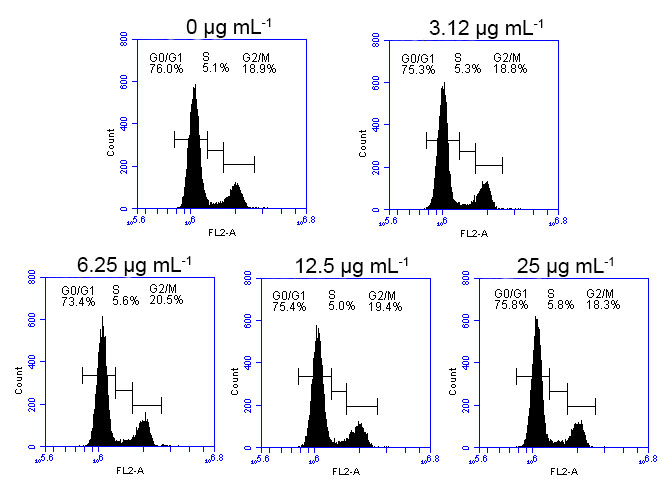


Fig.S4 Cell cycle analysis of BABL-3T3 cells treated with Fe-MIL-101 by flow cytometry.


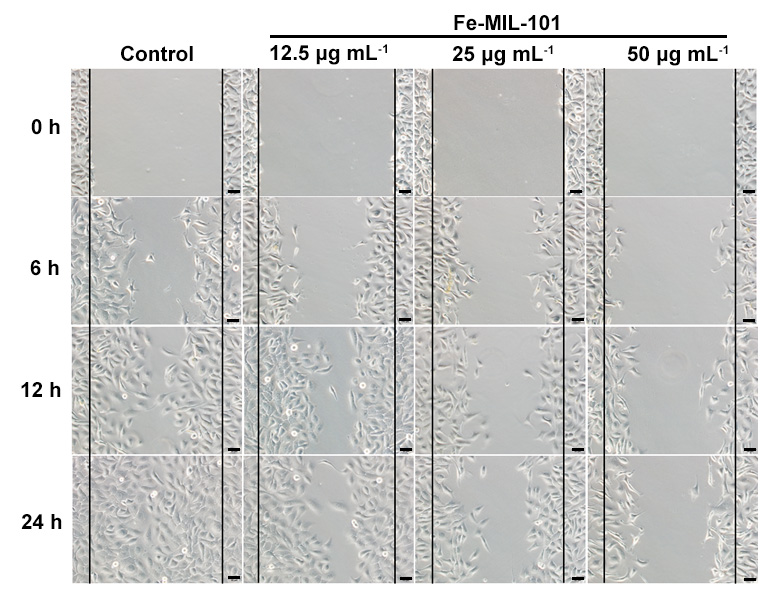


Fig.S5 Fe-MIL-101 inhibits SKOV3 cells migration by the wound-healing assay. Scale bar: 100 μm.

**References**

1. C. Tamames-Tabar, D. Cunha, E. Imbuluzqueta, F. Ragon, C. Serre, M. J. Blanco-Prieto and P. Horcajada, *J. Mater. Chem. B*, 2014, **2**, 262.
2. K. M. L. Taylor-Pashow, J. D. Rocca, Z. Xie, S. Tran and W. Lin, *J. Am. Chem. Soc.,* 2009, **131**, 14261.
3. R. C. Huxford, K. E. DeKrafft, W. S. Boyle, D. Liu and W. Lin, *Chem. Sci.*, 2012, **3**, 198.
4. W. J. Rieter, K. M. Pott, K. M. L. Taylor and W. Lin, *J. Am. Chem. Soc.*, 2008, **130**, 11584.
